# The ubiquitin–proteasome system and signal transduction pathways regulating Epithelial Mesenchymal transition of cancer

**DOI:** 10.1186/1423-0127-19-67

**Published:** 2012-07-24

**Authors:** Ioannis A Voutsadakis

**Affiliations:** 1 Centre Pluridisciplinaire d’Oncologie, BH06, Centre Hospitalier Universitaire Vaudois, Bugnon 46, Lausanne, 1011, Switzerland

**Keywords:** Epithelial to mesenchymal transition, Ubiquitination, Ubiquitin-proteasome system, Signal transduction, Carcinogenesis

## Abstract

Epithelial to Mesenchymal transition (EMT) in cancer, a process permitting cancer cells to become mobile and metastatic, has a signaling hardwire forged from development. Multiple signaling pathways that regulate carcinogenesis enabling characteristics in neoplastic cells such as proliferation, resistance to apoptosis and angiogenesis are also the main players in EMT. These pathways, as almost all cellular processes, are in their turn regulated by ubiquitination and the Ubiquitin-Proteasome System (UPS). Ubiquitination is the covalent link of target proteins with the small protein ubiquitin and serves as a signal to target protein degradation by the proteasome or to other outcomes such as endocytosis, degradation by the lysosome or specification of cellular localization. This paper reviews signal transduction pathways regulating EMT and being regulated by ubiquitination.

## Introduction

Epithelial to Mesenchymal transition (EMT) describes the process that allows an epithelial cell belonging in an epithelial membrane to detach from its neighbors, to transverse the dissolving basement membrane and move through the extra-cellular matrix to other sites of the tissue or even to distant organs. In order to facilitate mobility during EMT, connections joining the cell to adjacent epithelial cells are dissolved [1]. Concomitantly, the cell acquires a fibroblast-like shape, down-regulates epithelial markers and up-regulates mesenchymal markers. EMT is a process that physiologically takes place during development. Multi-cellular organisms derive their variety of specialized cells and tissues from a single cell, the fertilized ovum. This cell has to differentiate to the three layers of differentiation, the ectoderm, mesoderm and endoderm and further to different tissues and cell types in complex but ordered patterns. During development EMT takes place as an integral process of differentiation to the various cell types in a highly regulated in space and time manner. For example during embryonal gastrulation the epiblast layer produces a midline invagination, the primitive streak, from which cells are mobilized by undergoing an EMT and produce the mesoderm and endoderm. In another developmental example, dorsal neural tube-derived neural crest cells undergo an EMT and migrate to form components of the peripheral nervous system, skin melanocytes, adrenal medulla and facial bones and muscles [2].

Derived from a single cell, all cells of a multi-cellular organism possess the same DNA sequences in their whole genome and thus their various phenotypes must be the result of differences in transcriptional and post-transcriptional regulation of cellular proteins secondary to intra-cellular and external signals. Post-translational modifications can regulate function, localization and turn-over of every cell protein impacting on cell morphology, activity and interactions in the multi-cellular organism. Ubiquitination is such a post-translational modification. Ubiquitination is the covalent attachment of molecules of the small 76 amino-acids protein ubiquitin to a target protein which is then marked for proteasome destruction or endocytosis or participation in a range of processes. Ubiquitination along with other post-translational modifications of proteins such as phosphorylation, hydroxylation and acetylation is a regulated process for the execution of which a multitude of regulators exist. Many signal transducers and transcription factors involved in EMT are regulated by ubiquitination and the ubiquitin proteasome system. This review will discuss EMT signal transduction pathways and relationship with Ubiquitin-Proteasome system (UPS) while the extensive network of transcription factors regulating EMT and their relationship to UPS will not be discussed here.

### EMT in cancer

EMT is proposed to happen in three different scenarios with different starting points and outcomes. In development EMT is used by normal fetal cells for obtaining the different specificities present in the multi-cellular organism. In adult tissue injury repair, EMT is used to heal open wounds and may lead to fibrosis. In a third scenario, EMT happens during the tissue invasion and metastatic process of malignant epithelial cells. These three EMT types have recently been labelled by convention type 1, 2 and 3 [3]. The starting point of type 1 EMT is, thus, epithelial progenitors in the embryo that lose conduct with their initial site and move to become a different structure with different morphology and function. In type 2 EMT, epithelial cells of an injured site lose conduct with their neighbours to move and become fibroblasts contributing to injury repair but also possibly to pathologic fibrosis. Cancer associated EMT or type 3 EMT endows epithelial cancer cells with the ability to detach from their initial site, pass through the resolving basement membrane into adjacent tissues and even metastasize to distant sites. In vitro and in vivo criteria for the confirmation of EMT have been established and fall into two broad categories [4]. First, they include up-regulation of specific mesenchymal markers and down-regulation of epithelial-associated proteins. Second, they describe general cell properties of the new cell state. Mesenchymal proteins induced in EMT include S100A4 [also called FSP1(Fibroblast-Specific Protein 1)], vimentin, type I collagen and its receptor kinase DDR2 (Discoidin Domain Receptor tyrosine kinase 2), cadherin N and OB, transcription factors Snail1 and 2, ZEB1 and 2 and Twist, and nuclear localization of β-catenin. Down-regulated epithelial proteins include E-cadherin, ZO-1 (Zona Occludens 1), cytokeratins, claudins, occludins and basement membrane components collagen IV and laminin 1. General cell properties induced by the transition include a change in morphology with the acquisition of spindle shape, loss of epithelial cell polarity and stress fibers redistribution, resistance to apoptosis induction and enhanced migratory capability. Resistance to apoptosis in neoplastic cells undergoing EMT is accompanied by the acquisition of a stem cell phenotype also associated with drug resistance [5]. For the in vivo experimental confirmation of EMT the introduction of a cell reporter construct in epithelial cells that subsequently continues to be expressed in resulting mesenchymal cells has been proposed. Although the importance of EMT for cancer initiation and progression has been debated and even completely refuted [6], it becomes increasingly accepted as a program that promotes invasion and metastasis, a hallmark capability of cancer [7]. Part of the initial debate was due to semantic discrepancies and the difficulty to define EMT in vivo. Markers to serve this purpose have now been proposed and the situation is more clear with the realization that EMT in a cancer cell may not be complete and only part of the EMT markers may be expressed in each instance [4]. In the process of collective migration, for example, cells detach from the epithelial site, acquire mesenchymal properties but move en block without losing adhesions between them [8]. The acceptance of EMT as intrinsic to the malignant process has also been aided by the discovery that beyond specific EMT-inducing factors a multitude of general cancer regulating pathways are important EMT regulators as it will be discussed in following sections.

### Ubiquitination and the ubiquitin-proteasome system (UPS)

**Figure 1:**
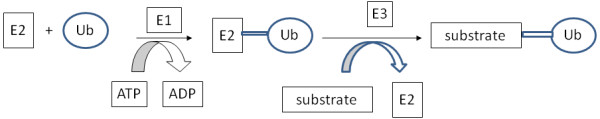
**The ubiquitination cascade.** E1 denotes ubiquitin-activating enzyme, E2: ubiquitin conjygating enzyme and E3 ubiquitin ligase. Ub: ubiquitin.

Ubiquitin attachment to a target protein is executed with the help of three types of enzymes. Initially an enzyme called E1 or ubiquitin activating enzyme binds ubiquitin through a thioester bond in an ATP and Mg^++^-dependent manner. Then ubiquitin is transferred to a cysteine residue of an ubiquitin conjugating enzyme or E2 as a thioester. E2-linked ubiquitin is finally transferred to a lysine residue of a target protein by an ubiquitin ligase or E3 (Figure 1). In the human genome there exist two E1 enzymes, about 30 to 40 E2 enzymes and about 600 or more E3 ligases [9].

The two major types of E3 ligases, RING (Really Interesting New Gene) type and HECT (Homologous to Human Papilloma Virus E6 Carboxyterminal domain) type, differ in their mode of catalysis but both result in ubiquitin ligation to the target protein. RING type E3s are by far more abundant than HECT E3s and comprise about 95% of human E3s [10], while HECT type E3s count 28 members in human genome [11]. Ubiquitination, like other post-translational modifications, is reversible and covalently-linked ubiquitin molecules can be removed by de-ubiquitinating enzymes which preserve cellular ubiquitin reserves and reverse inappropriate ubiquitination [12].

Ubiquitin is a 76 amino-acids protein that has lysine residues at positions 6, 11, 27, 29, 33, 48 and 63. Depending on the lysine that mediates attachment and the number of ubiquitin molecules that are attached to a target protein, this protein undergoes different fates. Lysine 48 ubiquitin chains of at least 4 molecules length lead to the recognition of the target protein by the proteasome and subsequent degradation [13]. Occasionally lysine 6 and 11-mediated ubiquitin chains have been observed to signal target protein proteasome degradation. Lysine 63-mediated ubiquitin attachment leads less often to proteasome degradation but serves mostly as signal for autophagy-mediated proteolysis, as well as non-proteolytic functions such as DNA repair and receptor kinases endocytosis. Other cell processes in which ubiquitination participates include DNA transcription and DNA damage tolerance.

The proteasome is a hollow barrel-shaped multiprotein structure of 2.5 MDa comprised of a core particle (CP or 20S proteasome) flanked in the two sides by a regulatory particle (RP or 19S proteasome). RP functions include recognition of the ubiquitinated protein, denaturing of the protein, de-ubiquitination which allows ubiquitin molecules to be recycled and delivery of the target protein to the CP. CP is made by four rings of seven member proteins each that are stuck one on the other. The two peripheral rings are identical and are called α rings (with sub-units α1 to 7) and the two central rings are also identical and are called β rings (with sub-units β1 to 7). The proteasome possesses three enzymatic activities, a trypsin-like (post-basic residues cleavage) activity, a chymotrypsin-like (post-hydrophobic residues cleavage) activity and a post-glutamyl (caspase-like or post-acidic residues cleavage) activity that reside in sub-units β1, β2, and β5 respectively and degrade target proteins producing fragments of 4 to 14 amino-acids [14].

### EMT signalling network

**Figure 2:**
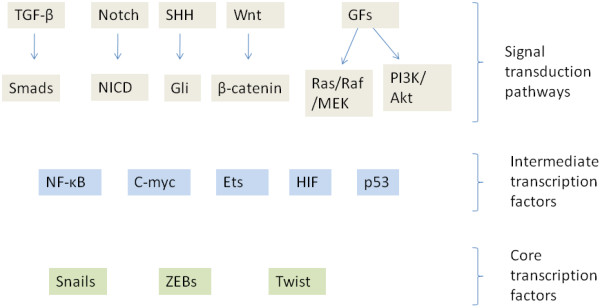
**A conceptual layer overview of cancer EMT.** Intermediate transcription factors induce or regulate core transcription factors and co-operate with them in target regulation culminating in down-regulation of epithelial genes and up-regulation of mesenchymal genes.

A survey of signalling pathways playing a role in EMT unsurprisingly reveals that they include most major pathways involved in carcinogenesis. These pathways signal through transcription factors which in their turn regulate and are regulated by transcription factors of the core EMT machinery such as Snail family regulators, ZEB and Twist (Figure 2). A discussion of pathways that regulate EMT follows. From this discussion the role of UPS in EMT regulation becomes evident.

**Figure 3:**
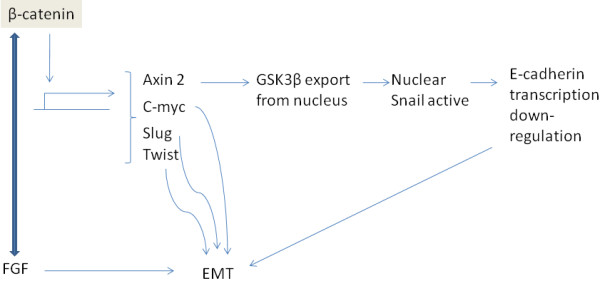
**Representation of EMT regulation by β-catenin.** β-catenin induces transcription of EMT-promoting proteins. Resulting adherens junctions dissolution makes available further β-catenin molecules for transcriptional activity except if ubiquitination and proteasomal degradation restrains this activity. Fibroblast Growth Factor (FGF) co-operates in β-catenin transcription (shown as a double edge arrow).

β-catenin, a transcription factor and adherens junction component, is controlled by the Wnt signalling pathway. The soluble ligand Wnt ligates its cell surface receptor Frizzled in complex with co-receptor LRP5/6 (Low density lipoprotein receptor-Related Protein 5/ 6) and activates intracellular protein Dishevelled (Dvl). Dvl inhibits protein kinase GSK3β preventing it from phosphorylating β-catenin. Unphosphorylated β-catenin is freed from the destruction complex (see below) to enter the nucleus and interact with transcription factors of the TCF 4 (T cell factor 4)/LEF (Lymphoid Enhancer Factor) family to initiate a transcription program leading to cell proliferation but also, depending on co-factors, to differentiation or maintenance of stem cell dedifferentiated phenotype [15]. In contrast, in the absence of Wnt, kinase GSK3β remains active and phosphorylates β-catenin, in co-operation with kinase CKIα (Casein Kinase Iα). A complex, called destruction complex, is formed with the participation of proteins axin, APC (Adenomatous Polyposis Coli) and the ubiquitin ligase β-TrCP which ubiquitinates β-catenin and leads to its proteasome degradation. Mutations in APC are seen in hereditary colorectal cancer syndrome Familial Adenomatous Polyposis and in the majority of sporadic colorectal cancer cases and result in constitutive activation of β-catenin due to failure of its ubiquitination. Among β-catenin target genes several involved in EMT are included such as c-myc. Axin2 is also induced by β-catenin and chaperones kinase GSK3β from the nucleus to the cytoplasm, an event that allows transcription modulator Snail to remain unphosphorylated and active to suppress E-cadherin transcription [16] (Figure 3). Snail2 (also known as Slug) and Twist are further regulated by Wnt signalling [17]. Moreover, other pathways such as FGF co-operate in Wnt pathway-induced EMT [18].

Transforming Growth Factor β (TGFβ) signalling is one of the best characterized pathways in carcinogenesis and a dual role is observed with a tumor suppressing effect during cancer initiation phase followed by a tumor-promoting effect associated with EMT in more advanced cancer when the Ras /PI3K/ akt pathway is often concomitantly activated and p53 is mutated or disabled [19]. Duration of TGFβ signalling and the cell cycle phase that the cell transverses may be additional determinants of TGFβ signalling outcome [20,21]. TGFβ is stored in the extra-cellular matrix in a latent form [22] and when released ligates its surface serine/ threonine kinase receptors TβRI and TβRII which then phosphorylate and activate Smad2 or 3 proteins. Phosphorylated Smad2/3 associates with Smad4 and the complex moves to the nucleus where it acts as transcription co-factor. TβRI possess further the ability to activate Ras through adaptor protein ShcA, and proteins Sos1 and Grb2 [23]. All backbone components of TGFβ cascade including TβRI and TβRII and Smads are regulated by ubiquitination and proteasome degradation [24]. Members of the Nedd4 (Neural precursor cells-expressed developmentally down-regulated 4) family of HECT E3 ligases such as Nedd4-2, Smurf1 and 2, WWP1 and Itch/AIP4 participate in this regulation [25]. TGFβ tumor suppressing or promoting influence is cellular context-dependent and is mechanistically determined by the presence of co-factors interacting with Smads mainly on target promoters leading to activation or suppression of different target gene sub-sets. For example, Ets (E26 Transforming Sequence) family transcription factor member EVI1 (Ecotropic Viral Integration site 1) antagonizes Smad3-mediated transcription after interaction with it and PPARγ activation also antagonizes Smad3-mediated E-cadherin suppression [26]. In contrast, Olig1, a bHLH (basic Helix Loop Helix) transcription factor co-operates with Smad2/3 complex in inducing PDGFβ (Platelet Derived Growth Factor β) transcription.

**Figure 4:**
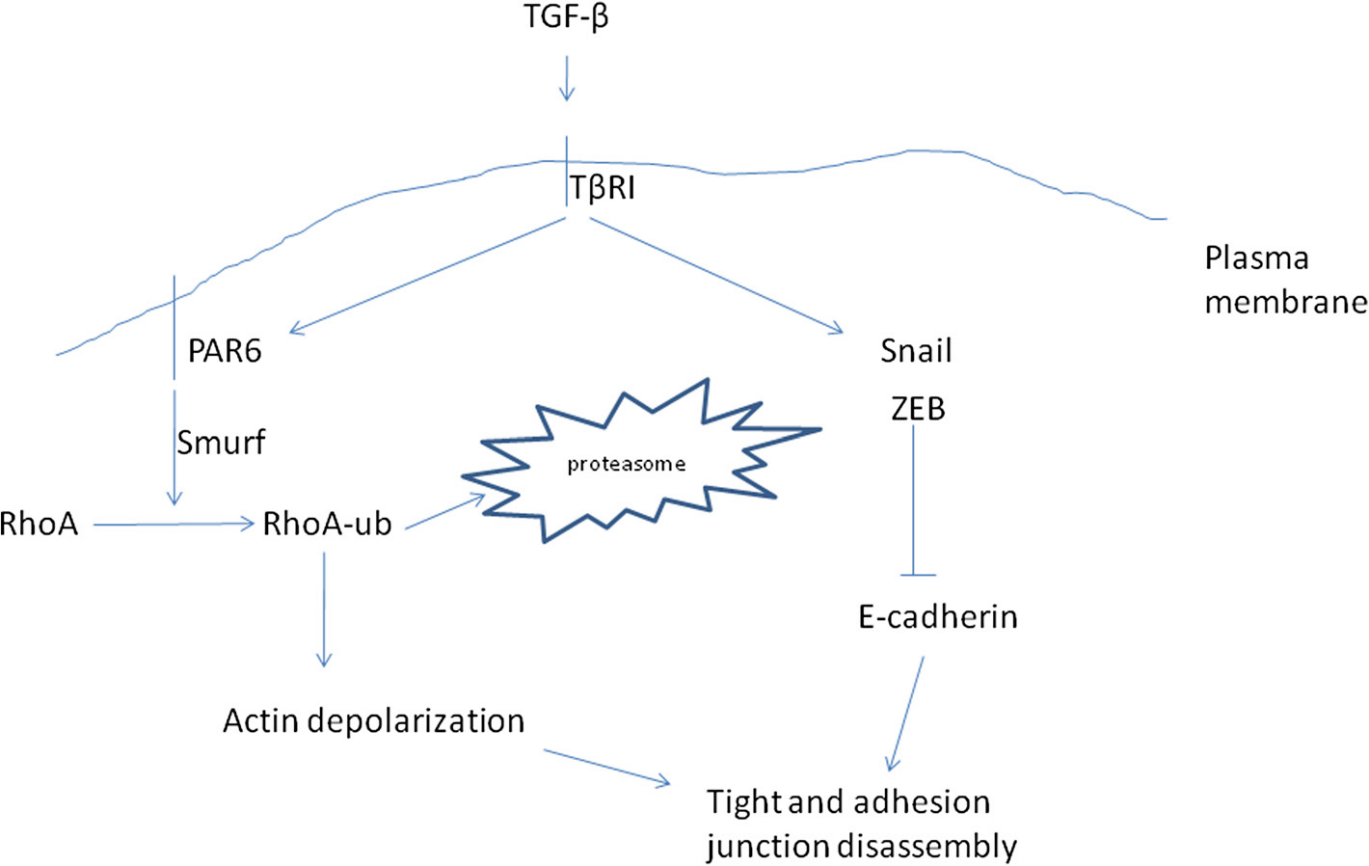
**Dual way of EMT regulation by TGF-β.** Activation of its receptors results in transcription activation through Smads and in transcription-independent direct effects on cellular junctions.

TGFβ promotes EMT by inducing Snail, Slug, ZEB1 and 2 and Forkhead factors FOXC2 and FOXQ1 resulting in E-cadherin suppression [27-31] (Figure 4). EMT promotion depends on the activation of the Ras pathway [32] and on neutralization of p53 by mdm2 induction [33].

TGFβ further promotes EMT by a transcription-independent way which functions through phosphorylation of the polarity complex protein Par6 by TβRI (Figure 4). Phosphorylated Par6 recruits E3 ligase Smurf1 promoting degradation of exchange factor RhoA and actin depolymerisation leading to tight junction disassembly [34].

A third signalling pathway important in both development and EMT involves surface receptor Notch. This receptor is activated after ligation to the ligand DSL (Delta/ Serrate/ LAG-2) in Drosophila, and Delta-like 1, 3 and 4 and Jagged-1 and 2 in mammals on the surface of a neighbouring cell [35,36]. Ligation of Notch by DSL results in extra-cellular cleavage of Notch near the external surface of plasma membrane by ADAM (A Disintegrin and Metalloprotease) proteases associated with ubiquitin-dependent endocytosis of DSL/ extra-cellular part of Notch into the signalling cell. DSL/ extracellular domain of Notch interaction uncovers the ADAM cleavage site and subsequent endocytosis clears extracellular domain of Notch from the inter-cellular space that could neutralize other DSL ligands from interacting with Notch molecules [37]. A γ-secretase complex cleaves the intracellular part of Notch (named Notch Intracellular Domain, NICD) from the transmembrane part (Intracellular and transmembrane part of Notch after ADAM action is called NEXT, Notch Extracellular Truncation) and NICD is freed to enter the nucleus and join transcription factor CSL [CBF1/ Su(H)/ Lag1 in Drosophila, named CBF1 (C promoter Binding Factor 1) or RBPJκ (Recombination signal binding protein for immunoglobulin kappa J region) in mammals] in transcription initiation. After NICD binding, RBPJκ is transformed from a transcription repressor to an activator and promotes transcription of the HES (Hairy and Enhancer of Split) family of bHLH transcription repressors [38]. Stability of NICD is regulated by an Fbxw7-containing SCF (Skp1/ Cul1/ F-box protein/ Rbx1) type E3 ligase which ubiquitinates it for proteasome degradation.

Notch activation after ligation by Jagged2 increases cancer cell survival in hypoxic conditions and promotes EMT [39,40]. This effect is associated with activation of Akt, Slug up-regulation and E-cadherin suppression. Akt activation by Notch depends on the transcription of a soluble factor that acts in an autocrine manner [41]. Although the exact nature of this factor is unknown, it could be one of the tyrosine kinase receptor ligands that are known to activate Akt. Induction of FGF2 mediated by Notch has indeed been documented in endothelial cells [42]. Additionally, HIF plays a role in Notch induced Snail and Slug up-regulation and EMT during hypoxia [43]. TGFβ/ Smad signalling promotes Jagged1-mediated Notch activation and EMT [44].

Another developmentally important pathway signalling in EMT is the one of Hedgehog (HH) ligating cell surface receptor Patched. Three Hedgehog ligands exist in mammals, Sonic, Indian and Desert Hedgehogs (SHH, IHH and DHH respectively). This ligation relieves the inhibition of protein Smoothened (Smo) by Patched. Smo, analogously to Dvl for the Wnt/ β-catenin pathway, inhibits phosphorylation of transcription factor Gli by kinases GSK3, CKI and PKA and frees the transcription factor to enter the nucleus and begin transcription. HH signalling promotes degradation by the ubiquitin-proteasome system of Gli inhibitor SuFu (Suppressor of Fused) [45]. In the absence of HH, Smo remains suppressed by Patched and phosphorylation of Gli by kinases GSK3, CKI and PKA allows its subsequent ubiquitination by a ubiquitin ligase complex in which β-TrCP participates and leads to its proteasome degradation. Furthermore, Nedd4 family ligase Itch is involved in Gli1 degradation [46].

**Figure 5:**
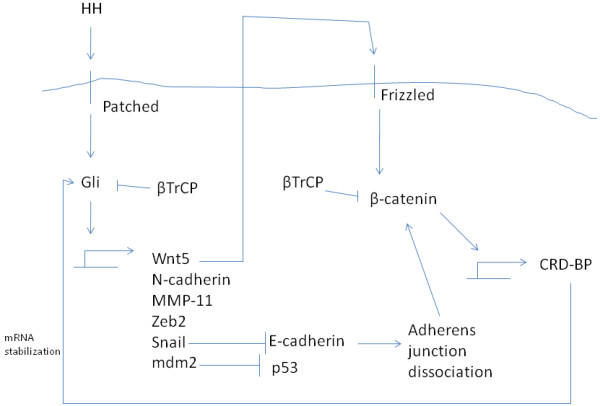
Interrelationship of hedhehog and wnt/ β-catenin pathways in EMT regulation.

Gli promotes EMT by activating transcription of ZEB2 in normal and neoplastic epithelial esophageal and gastric cells [47,48]. Several mesenchymal genes such as cadherin N are induced (Figure 5). Matrix Metalloproteinase 11 (MMP-11) is up-regulated by Gli signalling and facilitates EMT-associated invasion [49]. Wnt pathway is also induced by up-regulation of Wnt5A [48]. Reciprocally, β-catenin induces RNA binding protein CRD-BP which binds and stabilizes Gli mRNA up-regulating Gli signalling [50]. β-catenin was shown to be accumulating in the nucleus in endometrial carcinoma samples with activated Gli [51]. This effect may be related to the induction of Snail by Gli which suppresses E-cadherin and thus displaces β-catenin from adherens junctions [52]. In contrast, in colorectal carcinoma, Gli signalling has been associated with suppression of TCF transcription in more advanced stages and the interaction of the two pathways is complex [53]. Colorectal cancer cell lines transfected with Gli displayed decreased β-catenin transcriptional activity and patient samples showed a reverse relationship of Gli-β-catenin nuclear accumulation [54]. Activation of Gli depends also on the status of activation of the Ras/MAPK and PI3K pathways and of p53 which favour or inhibit Gli. There exists a reciprocal regulation of Gli on p53 because ligase E3 mdm2 is induced by Gli [55] (Figure 5). Besides Wnt and Ras/ MAPK, HH also interconnects with TGFβ in EMT regulation. HH signalling facilitates TGFβ-induced EMT which is reduced when HH is silenced by siRNA in lung carcinoma cells [56]. In addition, pharmacologic inhibition of HH in these cells decreased their migration and invasion [56].

Receptor tyrosine kinase (RTK) pathways including EGFR (Epidermal Growth Factor Receptor) family, PDGFR (Platelet Derived Growth Factor Receptor), c-met (the Hepatocyte Growth Factor Receptor), NGFR (Nerve Growth Factor Receptor) and FGFR (Fibroblast Growth Factor Receptor) are implicated in many carcinogenesis processes such as apoptosis inhibition, proliferation and angiogenesis as well as in EMT [57]. Two main downstream pathways are activated by these receptors, the Ras/ Raf/ MEK/ ERK pathway and the PI3K/ Akt pathway. The former culminates to activation of AP-1 (Activating Protein 1) family transcription factors while targets of PI3K/ Akt phosphorylation include apoptotic members of bcl-2 family which are inhibited, E3 ligases mdm2 and Fbw7 which are protected from inhibition, apoptotic transcription factor FoxO, kinase GSK3β, and caspase 9 which are all inhibited [58]. The mTOR cascade is also activated by Akt leading to enhanced cell growth and protein production but also EMT [59]. Implication in EMT is not surprising given the close relationship with other pathways such as Wnt/ β-catenin, TGFβ, NF-κB and p53. The JAK/STAT (Janus Kinases/ Signal Transducers and Activators of Transcription) pathway may also become activated by RTKs and activation of STAT3 transcription by EGFR signalling leads to Twist activation and EMT in breast cancer cells [60].

Receptor tyrosine kinase pathways are regulated by the UPS in multiple ways. Several core components of these pathways are proteasome substrates. Examples are kinases Raf [61], ERK1 and 2 [62] and ERK3 [63] of the Ras branch and the regulatory sub-unit p85 of PI3K [64] and kinase Akt [65] of the PI3K/ Akt branch. Additionally components of the JAK/ STAT pathway are UPS-regulated [66]. Tyrosine kinase receptors themselves are regulated by ubiquitination after ligand binding. Ligand binding induces ubiquitination with the aid of E3 ligase Cbl (Casitas B lineage Lymphoma) which then mediates clathrin-dependent receptor endocytosis through recognition by ubiquitin binding domains in clathrin associated proteins of clathrin-coated pits [67]. Receptor endocytosis may lead to receptor degradation or recycling to the cell surface in order to be available for further ligand interaction. In other instances signalling may even continue from internalized receptors in the early endosomes. Oncogenic mutations of RTKs may not only increase the activation of receptors but also promote their surface recycling [68].

Nerve Growth Factor Receptor TrkB induces EMT with concomitant Twist induction and suppression of E-cadherin [69]. EMT depends on Twist as Twist knockdown through RNA interference prevents it, and partially depends on Snail. Zeb1 plays an important role in TrkB-induced EMT and promotes metastasis and inhibition of anoikis probably down-stream of Twist and Snail [70]. Akt was also involved in TrkB-induced EMT in head and neck squamous carcinomas [71]. Other constituents of the PI3K pathway such as components of mTORC1 and 2, Raptor and Rictor and loss of phosphatase PTEN are involved in EMT [72,73]. Additional examples of RTKs promoting EMT include EGFR [74], Hepatocyte Growth Factor Receptor c-met [75,76], PDGFR [77] and FGFR [78].

Endothelin 1 (ET-1) signals through the Endothelin A Receptor (ET_A_R), a G-protein-coupled seven trans-membrane domain membrane receptor, in an autocrine and paracrine way. ET-1 signalling contributes to EMT by activating the PI3K axis, increasing Snail activity and down-regulating E-cadherin [79]. ET-1 and other neuropeptides are involved in progression of prostate cancer in its castration-resistant phase which is often chemotherapy-refractory [80]. Neuropeptides signalling is regulated by the UPS as both the PI3K axis and NF-κB signalling down-stream of them are UPS targets.

### GSK3 Kinase, a link between signalling pathways of EMT and the ubiquitin-proteasome system (UPS)

**Figure 6:**
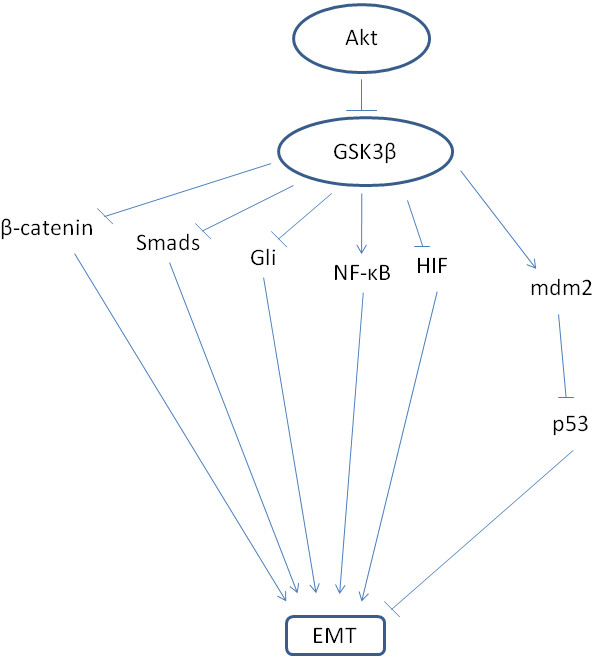
**Schematic representation of EMT regulation by serine/ threonine kinase GSK3β.** Phosphorylation by GSK3β is followed by ubiquitination and proteasome degradation of substrate proteins.

Serine/ threonine kinase GSK3 has multiple cellular substrates being one of the most versatile cellular kinases [81]. A major effect of substrates phosphorylation is subsequent ubiquitination. GSK3 regulates most of EMT involved pathways. In the Wnt pathway it promotes baseline β-catenin degradation until Wnt signalling inhibits it to allow β-catenin stabilization and transcription (Figure 6). In TGFβ family signalling, it promotes degradation of Smads to restrict their transcription activity. Both Smad1 and Smad3 are GSK3 phosphorylation targets and thus both the TGFβ and the BMP (Bone Morphogenic Protein) branches of the pathway are regulated in this manner [82,83]. In HH signalling, GSK3 promotes Gli degradation until ligand/ receptor interaction allows Smo to inhibit it and stabilize Gli for transcription.

Many intermediate transcription factors but also core transcription factors of EMT undergo regulation by GSK3 (Figure 6). NF-κB signalling is regulated by GSK3 at the level of co-factor Bcl-3. In addition the alternative NF-κB pathway is regulated by GSK3 through phosphorylation of precursor p105 which in this case leads to a partial proteasome-mediated cleavage producing the mature form NF-κB1 (p50) [84] and the canonical NF-κB activity is regulated by GSK3 phosphorylation of kinase IKK [85,86]. Protein RKIP, an NF-κB inhibitor, promotes GSK3β function, being an additional functional link between the two EMT regulators [87]. Transcription factor C-myc stability is regulated by phosphorylations by Akt and GSK3 followed by ubiquitination [88,89]. HIF1 is also a target of GSK3 phosphorylation which leads to VHL-independent proteasome degradation [90,91]. Mdm2 is a GSK3 substrate and its phosphorylation by the kinase results in activation of the ligase and p53 down-regulation [92]. Ionizing radiation promotes GSK3 phosphorylation at Serine 9 which inhibits activity towards Mdm2 and thus stabilizes p53. PTEN is another GSK3 substrate after a priming phosphorylation by kinase CK2 [93]. Finally, as mentioned, EMT regulating transcription factor Snail is a GSK3 substrate. GSK3 is regulated in the transcriptional level by transcription factor Ets2 down-stream of a mutant activated K-Ras [94].

As a result of its involvement in all these pathways, GSK3 represents a constitutive suppressor of EMT [95]. Its general mode of action requires a priming phosphorylation of the substrate by another kinase 3 to 4 aminoacids downstream from its own phosphorylation site, followed by phosphorylation by GSK3 [96]. This double phosphorylation sites are then recognized by E3 ligases for ubiquitination and proteasome degradation. Kinase Akt, an effector of receptor tyrosine kinase pathways is a GSK3 inhibitor and this is one of Akt actions promoting EMT (Figure 6). Nevertheless, there exist distinct cellular pools of GSK3 and not all of them can be inhibited by Akt. For example, the Axin associated GSK3 which keeps β-catenin under control is not regulated by Akt. This represents an additional level of complexity in the EMT-regulation network.

### E3 ligases regulating EMT

E3 ligases of both the RING and the HECT family participate in EMT regulation by ubiquitination. Several of these enzymes are known to regulate other carcinogenesis processes because pathways involved in EMT signalling play roles also in these processes and additionally because these ligases have further substrates that are involved in carcinogenesis beyond EMT. SCF (Skp1/ Cullin/ F-box) E3 ligases represent a sub-family of RING type ligases and several members are involved in EMT. Their general organization comprises a complex of several proteins and includes a cullin molecule which is the scaffold protein of the complex, an F-box protein that associates through a Skp (S phase kinase-associated protein) protein with cullin and binds the substrate to be ubiquitinated and a ROC (also called Rbx) RING finger protein that binds the complex through cullin and also binds the ubiquitin-loaded E2 enzyme [97]. The cullin protein undergoes neddylation, association with the ubiquitin-like protein NEDD8, an event that facilitates the assembly of the ligase complex and opens cullin configuration to promote E2 enzyme binding.

βTrCP, a RING type E3 ligase of the SCF sub-type regulates EMT through its involvement in Wnt, HH and NF-κB cascades and in Snail degradation. βTrCP recognizes phosphorylated substrates and thus the process of target destruction is tightly regulated by at least one and sometimes two steps of phosphorylation followed by ubiquitination. In the Wnt pathway βTrCP inhibits signalling at base line conditions by degrading β-catenin when this protein is successively phosphorylated by casein kinase I and GSK3. In HH signalling, βTrCP participates in the ubiquitination of Gli when the pathway is silent. In both these pathways kinase GSK3 performs a pre-required phosphorylation step. Proto-oncogene H-Ras is also a target for βTrCP ubiquitination [98]. As a result, βTrCP has both promoting and inhibiting roles in EMT.

Skp2 is the F-box component of another SCF family RING E3 ligase and is involved in carcinogenesis processes through regulation of Cyclin Dependent kinase inhibitors p27 and p21 [99]. Skp2 promotes TGFβ signalling, contributing to EMT regulation. It co-operates also in degradation of mutated non-functional Smad4 promoting TGFβ dependent signalling [100]. This signalling reciprocally promotes Skp2 nuclear localization where it is ubiquitinated by E3 ligase APC/C (Anaphase Promoting Complex/ Cyclosome) for degradation [101].

Fbw7 (F-box and WD repeat domain-containing 7, also designated Fbxw7 or hCdc4), a third SCF E3 ligase regulating signal transduction of EMT does so through its role in degradation of Notch. C-myc is a transcriptional target of Notch and thus it is regulated also indirectly by Fbw7 as the ligase targets both Notch and presenelin, a component of its activating enzyme γ-secretase [102].

Fbw7 mutations are synergistic with p53 mutations in cancer induction in experimental models, given that p53 represents a safeguard mechanism of unopposed c-myc activity. This is a developmentally preserved mechanism as, in hematopoiesis for example, Fbw7 preserves hematopoietic stem cells quiescence while its deletion results in transient cell growth due to c-myc and cyclin E (another Fbw7 target) over-activity but finally to stem cells exhaustion due to p53-induced apoptosis [103]. Analogously, in carcinogenesis concomitant Fbw7 and p53 mutations would lead to unopposed EMT.

An additional member of the SCF family of ligases regulating EMT pathways is Cullin 7/ Fbxw8 [104]. Skp1 participates in the ligase complex associating Cullin 7 with Fbxw8 and ROC1 is the E2 recognizing unit. Cullin 7/ Fbxw8 has IRS1 (Insulin receptor substrate 1), a mediator of IGF (Insulin-like Growth Factor) pathway, as a substrate [105]. This pathway, as other RTK pathways, activates the Ras/ Raf/ MEK/ ERK and the PI3K/ akt cascades and thus may play a role in EMT in cells that express the receptor. In addition, cullin 7/ Fbxw8 is further implicated in EMT regulation by interfering with p53 function in a degradation- independent manner [106].

NEDD4 family of E3 ligases belong to the HECT type ligases and have nine members in humans, several of which regulate EMT through their role in TGFβ signalling. All members of the family have three types of conserved domains. In their amino-terminal part they have a calcium binding domain designated C2 which mediates membrane localization after calcium binding. In the central part of their molecule NEDD4 ligases harbour two to four WW domains which recognize substrates and in the carboxy-terminal end they have the HECT catalytic domain which has an active cysteine that binds ubiquitin with a thioester bond before transfer to the substrate [107]. NEDD4 ligases are regulated by phosphorylation which may be either activating or inhibitory depending on the site of the phosphorylation [108,109] and by auto-ubiquitination [110]. Beyond TGFβ signalling NEDD4 ligases are involved in EMT by regulating other pathways. Among these are RTK pathways through ubiquitination of several proteins such as receptors themselves or down-stream components. NEDD4 promotes degradation of Vascular Endothelial Growth Factor Receptor 2 (VEGFR2) [111] and of IGF-1R [112]. NEDD4L targets the nerve growth factor receptor TrkA [113]. Cbl family ligases that regulate receptors endocytosis and recycling/ degradation are targets in their turn of NEDD4 family members-mediated ubiquitination [114,115]. Phosphatase PTEN, an inhibitor of the PI3K/ Akt cascade is ubiquitinated by NEDD4 family members and this modification modulates both its stability and sub-cellular localization [116-118].

Cbl is a RING E3 ligase that contributes to cell motility and EMT. Two main mechanisms mediate the influence of this ligase on EMT. The first is by regulating signalling of cell surface receptors which, as mentioned, are ubiquitinated and endocytosed after ligand binding. The second mechanism involves the targeting of adhesion-related molecules such as kinase FAK and integrin α5 by Cbl. Cbl also interferes with the stability of actin cytoskeleton through its involvement in the ubiquitination of proteins mDab1 (mouse Disabled homologue 1) and WAVE2 (Wiskott-Aldrich syndrome protein verprolin homology 2), proteins participating in G-actin polymerization [119,120].

## Conclusion

This review aims at providing a general overview of the complexity that underlines the relationship of EMT with ubiquitination focusing on major UPS regulators of EMT signal transduction pathways. Examples of common patterns of UPS regulation in different pathways of EMT are also provided as well as the inter-connections of these pathways mediated by UPS components.

Signalling inducing EMT overlaps significantly with pathways ensuring other carcinogenesis processes such as proliferation, inhibition of apoptosis, self-sufficiency for survival and invasion [7]. In addition EMT phenotype association with stem cell phenotype [5] and drug resistance has been increasingly recognized, implying that all carcinogenesis processes are inter-related and emblazoned into the same hardware. Numerous inter-connections ensure the tight control of signals in physiologic conditions and the UPS is an integral part.

Of particular interest is the connection of EMT signalling with cell polarity and asymmetric cell division regulation in view of the cancer stem cell theory and the relationship of EMT and stemness. In this respect cancer may be modelled as a disease of increased symmetric stem cell divisions leading to increased number of neoplastic stem cells. Regulators of EMT such as TGFβ and p53 are shared with asymmetric cell division and this may have implications for cancer pathogenesis and intrinsic ability of metastasis [34,121]. The aforementioned transcription-independent regulation of Par6 by TGFβ signalling, besides promoting EMT, also promotes loss of cell polarity by down-regulating Par complex component Par3 and by displacing the complex from the apical part of the cell membrane to the cytoplasm [122,123]. Cell polarity is important for asymmetric cell divisions because it helps ensuring the asymmetric inheritance of proteins determining cell function and fate by the two daughter cells. An example illustrating this point is represented by adaptor protein Numb which in asymmetric divisions segregates in the daughter cell that is devoid of membrane Par complex. Numb promotes degradation of Notch and Gli by interacting with E3 ligase Itch and, as a result, the cell that inherit Numb displays decreased activity of Notch and HH pathways which are important determinants of stem cell fate but also promoters of EMT [124]. In addition, Numb promotes stability of p53 by inhibiting ligase Mdm2 [125]. It is evident that all these functions suppressing EMT would be lost if asymmetric division is perturbed. In this case, as a result of collapsed adhesions and polarity, cells would be left with modified signalling that would favour motility and metastasis. The implication of these events in carcinogenesis is that the same cancer-promoting signals that favour proliferation of cancer stem cells endow these cells with an inherent metastatic potential by disabling normal control on EMT promoting pathways.

## Competing interest

The author confirms that there are no conflicts of interest related to this article.

## Authors’ contributions

IAV solely contributed to the conception, literature review and writing of the article.
